# Triple A syndrome with a new mutation pattern, first documented case in Austria: a case report with literature review

**DOI:** 10.1186/s13023-026-04465-5

**Published:** 2026-07-07

**Authors:** Karolina Anderle, Theresa Muellner-Bucsics, Alek Stadlmann, Clemens Gangl, Franco Laccone, David Horner, Safoura Sheikh Rezaei, Michael Trauner, Christian Mueller

**Affiliations:** 1 Department of Clinical Pharmacology, Medical University of Vienna, Vienna, Austria; 2 Division of Rheumatology, Department of Internal Medicine III, Medical University of Vienna, Vienna, Austria; 3 Division of Gastroenterology and Hepatology, Department of Internal Medicine III, Medical University of Vienna, Vienna, Austria; 4 Division of Cardiology, Department of Internal Medicine II, Medical University of Vienna, Vienna, Austria; 5 Institute of Medical Genetics, Medical University of Vienna, Vienna, Austria

**Keywords:** Triple A syndrome, Allgrove syndrome, AAAS gene, ALADIN protein, Novel nonsense-mutation, Achalasia, Adrenal insufficiency, Alacrima

## Abstract

**Purpose:**

Triple A syndrome, also known as Allgrove syndrome, is a rare autosomal recessive disorder, characterised by achalasia, alacrima, and adrenal insufficiency. The syndrome is caused by various mutations in the AAAS gene, encoding the protein ALADIN, leading to many heterogeneous clinical manifestations.

**Methods:**

We conducted a case-based literature review. The patient gave his informed consent prior to his inclusion in this study. All personal data was anonymised. This study was approved by the Ethics committee of the Medical University of Vienna and performed in accordance with the Declaration of Helsinki.

**Results:**

We report a case of a 51-year-old male patient with recurring symptoms of dysphagia due to achalasia, who was previously treated by Heller myotomy and repeated endoscopic dilations. He was diagnosed with primary adrenal insufficiency in childhood and substituted with fludrocortisone and hydrocortisone. The Schirmer test confirmed total alacrima, and neurological testing showed mild peripheral and central neuropathy. Genetic analysis was performed using PCR-amplification of the AAAS coding exons followed by sequencing. Long-read sequencing confirmed compound heterozygosity for two point mutations in the AAAS gene: a missense mutation, ENST00000209873.9:c.938T>A (p.Val313Asp) in exon 10, and a novel nonsense mutation, ENST00000209873.9:c.1264C>T (p.Gln422Ter) in exon 14.

**Conclusion:**

This is the first documented case of Triple A syndrome in Austria. Notably, some of the patient’s characteristic symptoms presented unusually late in life. By utilising long-read sequencing, we experimentally confirmed the in trans orientation of a new mutation located at a previously described position and a novel nonsense variant. We strongly recommend genetic testing for patients presenting with at least two symptoms of the Triple A triad (achalasia, adrenal insufficiency, alacrima) or associated neurological symptoms, as clinical manifestations can be delayed.

## Introduction

Triple A syndrome, also known as Allgrove syndrome, is an autosomal recessive disorder, characterised by adrenal insufficiency due to adrenocorticotropic hormone (ACTH) resistance, alacrima, and achalasia and was first described in 1978 [1]. Besides these cardinal symptoms, Triple A syndrome might also cause neuropsychiatric symptoms, such as peripheral and central neuropathy, mental retardation, or dementia. Moreover, it can lead to short stature and osteoporosis. Some patients also develop dermatological features such as palmo-plantar and punctate hyperkeratosis, and delayed wound healing or oral symptoms including xerostomia, angular cheilitis, glossitis, fissured tongue or even a premature loss of permanent teeth [2, 3]. Several clinical cases with different mutations have been reported in the literature [4–6]. However, the exact genotype-phenotype correlation of this ubiquitously expressed gene has not been established yet.

The complex expression and molecular involvement of the ALADIN (alacrima-achalasia-adrenal insufficiency-neurologic disorder) protein explain the multi-systemic character and the broad variation in symptoms and disease severity. To this end, we want to present the first patient with a novel mutation in the achalasia-addisonianism-alacrima syndrome (AAAS) gene worldwide, who is also the first patient with the Triple A syndrome in Austria.

### Case report

We describe a case of a 51-year-old male patient with longstanding recurring dysphagia of solid food and fluids and vomiting who was referred to the Division of Gastroenterology and Hepatology of the Medical University of Vienna. The patient gave his informed consent prior to his inclusion in the study. This study has been approved by the Ethics committee of the Medical University of Vienna and has been performed in accordance with the Declaration of Helsinki.

**Fig. 1:**
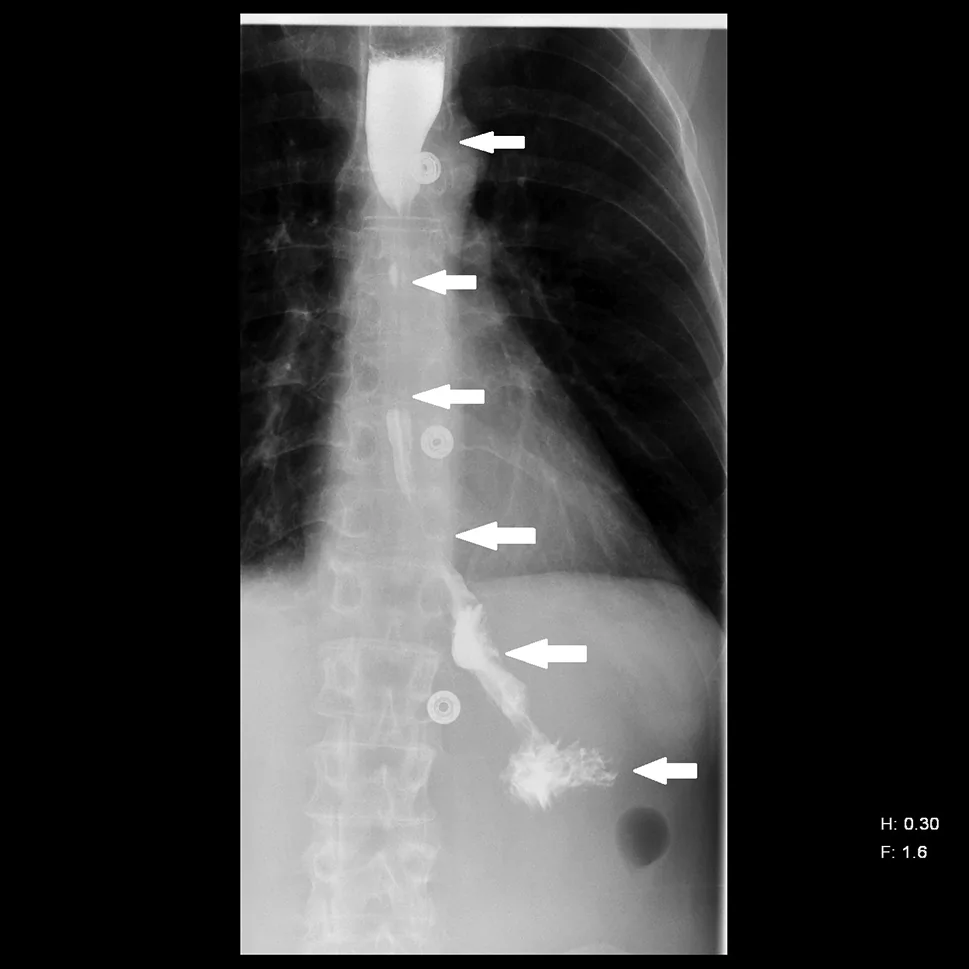
Barium swallow radiograph demonstrating a dilated and tortuous oesophagus (arrows)

A barium swallow test showed dilation of the proximal oesophagus, narrowing of the distal end and tertiary contractures (Fig. 1), which were then successfully treated with endoscopic dilation. The patient was under glucocorticoid and mineralocorticoid replacement therapy due to adrenal insufficiency since the age of seven. His plasma ACTH, renin, serum electrolyte, and aldosterone levels were found to be within normal ranges. Upon taking the medical history, dysarthria was noticeable. The patient reported the absence of tears since early childhood, which was confirmed by the Schirmer test.

**Fig. 2:**
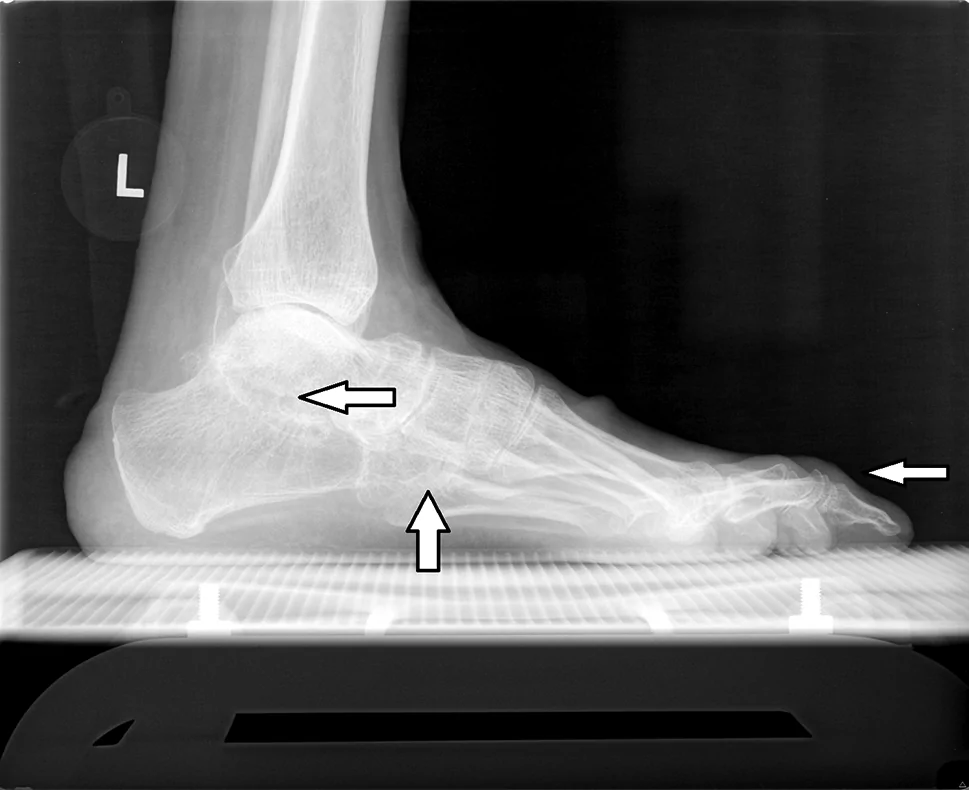
Pes cavus with degenerative arthritis of the talocalcaneal joint and a hammer toe deformity (arrows) in a lateral standing radiograph of the left foot

Physical examination revealed fissured, dry, and atrophic tongue, and palmoplantar keratosis. His gait was clumsy and shuffling, and he had cavoid feet with peripheral neuropathy (Fig. 2). Further examinations revealed muscle atrophy in the distal upper and lower extremities with slight to moderate spasticity and exaggerated reflexes in lower extremities with clonus, positive Babinski sign, and ataxic responses to tandem gait and finger-to-nose test. The patient also showed pyramidal and cerebellar signs and mild intellectual disability. Computed tomography disclosed a discreet global cerebral atrophy, while the cerebral magnetic resonance imaging illustrated atrophic glandulae lacrimales. An 18 F-FDG-PET/CT-scan revealed fructose-1,6-diphosphate (FDP) hypometabolism in the parietal, postcentral and frontomesial regions. Conversely, an electrocardiogram and a hearing test did not show any abnormalities.

### A long way to diagnosis

As a four-year old, the patient suffered from episodes of loss of consciousness due to hypoglycaemic events as well as from fatigue, abnormal skin pigmentation and hypotension. Primary adrenal insufficiency was diagnosed by an ACTH stimulation test, and he was started on hydrocortisone and fludrocortisone at the age of seven. Relapsing episodes of hypotension, fatigue, and weight loss were reported at the age of 18, which were caused by insufficient glucocorticoid supplementation. Over time, he developed progressive osteopenia on both lower extremities. The patient presented with unclear episodes of fever and flank pain at the age of 23. Laboratory investigations including immunological screening, viral serology testing, and tests for autoantibodies against adrenal tissue were performed but were found to be negative. Clinical suspicion of tuberculosis with adrenal gland involvement was raised and he was empirically started on anti-tuberculosis medication with a four-drug combination therapy. This tentative diagnosis, however, was never confirmed by radiologic, histological, or laboratory findings.

By the age of 29, a progressive degenerative joint disease emerged initially in distal lower extremities, later in the upper extremities. Multiple arthrodesis surgeries in upper and lower extremities with delayed wound healing were reported. Recurring pain episodes in the extremities resulted in the diagnosis of complex regional pain syndrome.

The patient’s gastrointestinal symptoms including dysphagia and vomiting started at the age of 40. Upper gastrointestinal endoscopy showed impaired oesophageal motility with stenosis of the gastro-oesophageal junction, corresponding to the diagnosis of achalasia. After two unsuccessful pneumatic dilation attempts, the patient underwent Heller myotomy with an anterior hemiplication at the age of 41. As the symptoms of achalasia did not resolve, another gastro-oesophageal dilation attempt followed at the age of 49.

Also at the age of 41, reduced sensory and motor conduction velocities in the peroneal nerve, reduced motor responses and chronic denervation changes were observed, consistent with a widespread peripheral neuropathy. Brain magnetic resonance imaging (MRI) was normal. Spine MRI showed a disc protrusion without a cystic cavity within the spinal cord. Around the same time, the patient was tested negative for 21-hydroxylase deficiency. His family medical history was unremarkable for gastroenterological, neurological, hormonal or metabolic diseases or any other medical conditions. As reported by the patient, his non-consanguineous parents did not have similar symptoms, and he did not have siblings.

### Molecular genetic testing

**Fig. 3:**
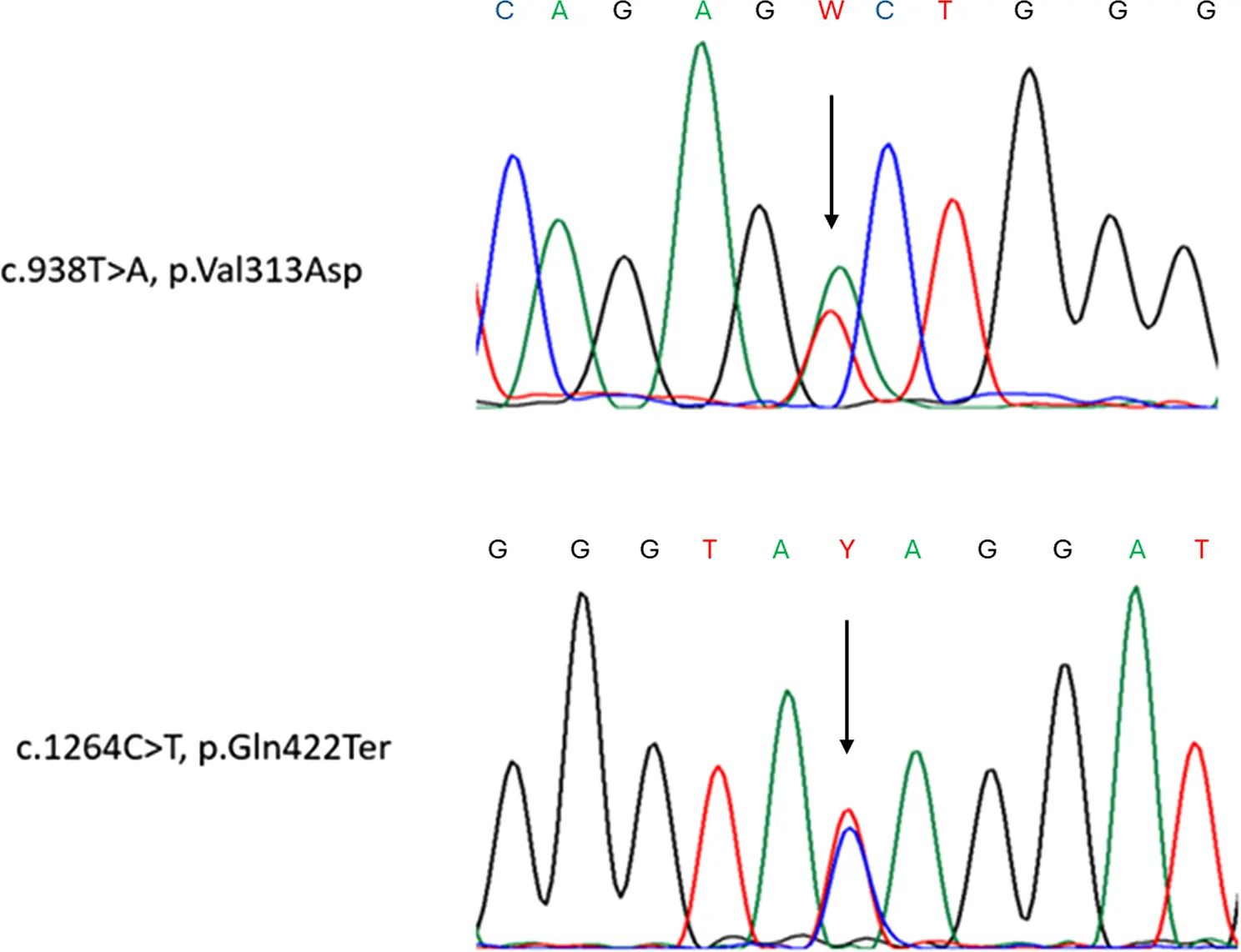
Sanger sequencing chromatograms depicting two heterozygous mutations. **(A)** The top chromatogram shows a heterozygous missense mutation, c.938T > A, resulting in a valine-to-aspartic acid substitution at amino acid position 313 (p.Val313Asp). The overlapping red (T) and green (A) peaks at position 938 indicate heterozygosity. **(B)** The bottom chromatogram illustrates a heterozygous nonsense mutation, c.1264 C > T, which leads to a glutamine-to-stop codon substitution at amino acid position 422 (p.Gln422Ter). The superimposed blue (C) and red (T) peaks at position 1264 confirm the heterozygous nature of this mutation. In both panels, the arrows indicate the position of the nucleotide changes

Since the clinical presentation with achalasia, alacrima and adrenal insufficiency suggested the presence of a Triple A syndrome, genetic testing by polymerase chain reaction (PCR)-amplification of the coding exons of the AAAS gene and subsequent sequencing was performed. We detected compound heterozygosity for two point mutations in the AAAS gene, a missense mutation ENST00000209873.9:c.938T > A, (p.Val313Asp) at a previously described position [6] in exon 10 and a novel nonsense mutation: ENST00000209873.9:c.1264 C > T, (pGln422Ter) in exon 14 (Fig. 3).

The compound heterozygosity of the two variants was confirmed by long-range PCR long-read sequencing. The region of interest in the AAAS gene was amplified using the following primers: AAAS_Ex10-14_F (5’-CCA CTA GAA GAA AAG GTG AGC AGG-3’) and AAAS_Ex10-14_R (5’-CTA CCA CTC CTT CAG CTG TCT TTC G-3’), and the PCR-product was sequenced using the SQK-NBD114.24 Kit from Oxford Nanopore. The segregation of the read sequences based on nucleotide composition at the genomic positions NC_000012.12:g. 53,308,119 and NC_000012.12:g.53,309,018 confirmed the in-trans position of the variants (Table 1).

**Table 1:**
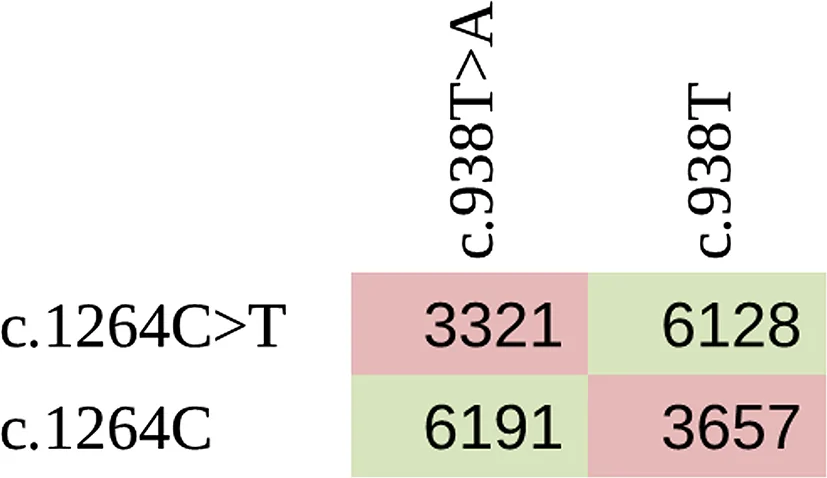
Table listing the read distribution of reference and/or variant combinations at the positions listed above. In green are allele combinations of the patient, while in red the crossprimed PCR artifacts

The affected AAAS gene, located on chromosome 12q13 [7], codes for the protein “alacrima, achalasia, adrenal insufficiency and neurologic disorder” (ALADIN), which consists of 546 amino acids. This protein contains the short structural motif terminating in a Trp-Asp (WD) dipeptide (WD-repeat). This peptide exhibits a high degree of functional diversity including regulation of signal transduction and RNA processing and transcription. To date, a total of 83 variants of the AAAS gene were classified as pathogenic and 33 as likely pathogenic; among these, 32 were frameshift variants (small insertions/deletions), 26 were nonsense variants, 13 were missense variants, and 30 affected splice sites [8].

### Discussion and review of literature

We report the first diagnosed patient with Triple A syndrome in Austria. Notably, we also found compound heterozygosity for a known and a novel mutation in the AAAS gene. His clinical presentation encompassed a wide range of symptoms and findings, which were in accordance with previously published cases, although some symptoms manifested later than typically described.

The Triple A syndrome, also called Allgrove syndrome (OMIM #231550) is a rare monogenetic but multi-systemic disease, which is characterised by a triad of symptoms comprising primary adrenal insufficiency, esophageal achalasia, and alacrima [1]. Alternative nomenclatures for this condition can be found in the literature. It is sometimes referred to as a 4 A syndrome if it is accompanied by autonomic dysfunction and progressive neuropathy [9] or 5 A syndrome in presence of (bulbospinal) amyotrophy and autonomic neuropathy [10]. The term “Double A syndrome” can be used when only two of the main features are present [11], which occurs in around one third of the patients [12]. Additionally, dermatological and oro-dental manifestations, osteoporosis, optic atrophy, and morphological abnormalities like short stature, microcephaly, and other dysmorphic features were reported [13].

The multi-systemic phenotype can be explained by the ubiquitous presence of the nuclear pore scaffolding protein ALADIN, which is mutated in affected patients. This protein helps prevent DNA damage from oxidative stress by importing DNA repair proteins (aprataxin and DNA ligase I) and ferritin heavy chain (FTH1) into the nucleus. A disrupted redox homeostasis, i.e. by faulty ALADIN proteins, can lead to cellular damage in highly oxidative tissues, including neuronal cells, fibroblasts, and adrenal cells [4, 14–18].

The inheritance pattern of the Triple A syndrome is autosomal-recessive, and many diagnosed patients have consanguineous parents [15]. A clear genotype-phenotype correlation or sex differences have not been established yet [12].

To date, around 83 different pathogenic mutations of the AAAS gene have been described. Based on geographical distribution patterns and migration studies, common ancestors were suggested in respective genotypes. One of the most frequent ones is the c.1331 + 1G > A mutation, which was documented worldwide and is predominant in North Africa and the Middle East [16, 19–21]. Its first appearance was estimated to date back 47 generations [19, 22]. Another frequently described genetic mutation p.Ser263Pro, mostly found in Europe in patients of Slavic origin [15, 23]. Our patient has confirmed compound heterozygosity for two point mutations, one of which is a novel nonsense mutation: ENST00000209873.9:c.1264 C > T, (pGln422Ter). The other is a missense mutation in a previously described location for pathogenic missense mutations: ENST00000209873.9:c.938T > A, (p.Val313Asp) [6, 24] 

Since its first description by Allgrove et al. in 1978, fewer than a hundred Triple A patients have been reported in the literature. As a result of the high phenotypical variability, the overall prevalence of the Allgrove syndrome might be fairly underestimated. Disease manifestation and diagnosis usually occur in childhood and reports of newly diagnosed adult patients are rare [25].

Achalasia is one of the main clinical features of the Triple A syndrome and usually presents with episodes of nausea, vomiting, and dysphagia. It can be diagnosed with esophageal manometry and barium swallow X-ray. Depending on severity, achalasia can be treated surgically, i.e. with laparoscopic Heller myotomy with a partial fundoplication, or endoscopically by botulinum toxin injections, pneumatic dilation or peroral endoscopic myotomy (POEM) [28]. In contrast to previously reported adult cases [26, 27], our patient suffered from late onset progressive achalasia after the age of 40, for which he underwent multiple oesophageal dilations and Heller myotomy.

Alacrima appears to be the most common clinical feature of Triple A syndrome and presents with decreased or absent tear production in early infancy prior to other manifestations, which is congruent with the disease course in our patient.

According to published cases, adrenal insufficiency is generally diagnosed in the first two decades of life but does not appear to be inborn [23, 29]. It first manifests as an adrenal crisis due to ACTH resistance is diagnosed based on hypocortisolaemia, hypoglycaemia, hypotension, elevated plasma ACTH levels and a negative ACTH stimulation test, which was the case in our patient who suffered from hypoglycaemic events, fatigue, skin pigmentation, and hypotension since he was 4 years old, which is in line with the literature [30]. The treatment consists of substitution of hydrocortisone, and in patients with mineralocorticoid deficiency also with fludrocortisone.

Neurological disorders are the most variable symptoms in adult patients with Triple A syndrome, encompassing limb weakness due to amyotrophy, axonal neuropathy with motor, sensory or sensorimotor involvement, pyramidal signs, cerebellar ataxia, dysarthria, hyperreflexia, parkinsonian sign and/or seizures, and, most characteristically, peripheral neuropathy [31–36]. The onset of his neurological symptoms appeared in advance of achalasia, which is atypical when compared to reported adult cases [9, 26]. Biopsies of the peripheral nerves performed in almost a dozen patients with Triple A syndrome showed unremarkable findings in some patients and a variable degree of axonal degeneration with demyelinated and unmyelinated axons in others. Hence, clinical features like fatigue or muscle weakness might be interpreted as a neurological manifestation in patients with Triple A syndrome [37]. Moreover, progressive bulbospinal amyotrophy in Triple A syndrome can also mimic amyotrophic lateral sclerosis [32, 38, 39]. Mild intellectual disability as well as delayed wound healing, which were diagnosed in our patient, have also been previously reported in Triple A syndrome [13, 40].

We assume that, due to the delay of onset and the varying presence of characteristic clinical features in Triple A patients such as in ours, many cases may remain unrecognised. Additionally, there is a wide spectrum of other diseases associated with congenital lacrimal secretory disorders like familial dysautonomia (which also comprises gastroesophageal and neurological symptoms) (41–43) or anhidrotic ectodermal dysplasia [44]. Some genetic diseases may even mimic Triple A syndrome, such as the AAMR syndrome that comprises alacrima, achalasia and mental retardation but no adrenal insufficiency [45–47], while patients with familial glucocorticoid deficiency disorders may also present with primary adrenal insufficiency, growth defects and neurological phenotypes at varying ages [48, 49]. Therefore, we recommend initiating genetic testing and a comprehensive multidisciplinary diagnostic evaluation in patients presenting with at least two phenotypic features [50].

## Conclusions

This patient is, to the best of our knowledge, the first patient diagnosed with Triple A syndrome in Austria. Importantly, he is the first patient with this specific constellation of two compound heterozygotic mutations in AAAS gene worldwide, a missense mutation on position 938 (T > A) and a novel nonsense transition mutation on position 1264 (C > T). Furthermore, the occurrence of neurological symptoms at the age of 29 and late-onset achalasia symptoms at the age of 40 reflect a rather atypical, protracted course of this disease leading to its late diagnosis.

Thus, we recommend testing patients for Triple A syndrome when presenting with at least two of the cardinal features including adrenal insufficiency, alacrima, achalasia and possible copresence of neurological symptoms, regardless of the time of symptom onset.

## Data Availability

All relevant data and materials are included in the manuscript. More detailed information can be provided upon reasonable request.
